# Drug induced Kounis syndrome: does oxidative stress play a role?

**DOI:** 10.1186/s12948-018-0099-2

**Published:** 2018-10-01

**Authors:** Luisa Ricciardi, Fabiana Furci, Marco Casciaro, Eleonora Di Salvo, Mariateresa Cristani, Valeria Tigano, Paola Lucia Minciullo, Sebastiano Gangemi

**Affiliations:** 1 Allergy and Clinical Immunology Unit, Department of Clinical and Experimental Medicine, University of Messina, AOU Policlinico “G.Martino” Via Consolare Valeria 1, 98124 Messina, Italy; 2 Messina Unit, National Research Council of Italy (CNR), Institute of Applied Science and Intelligent System (ISASI), Messina, Italy; 3 Department of Chemical, Biological, Pharmaceutical and Environmental Sciences, University of Messina, Messina, Italy

**Keywords:** Kounis syndrome, Cardiac anaphylaxis, Drug allergy, Oxidative stress, Advanced oxidation protein products, Advanced glycation end products

## Abstract

**Background:**

Kounis syndrome (KS) has been described as the coincidental occurrence of acute coronary syndromes during an allergic reaction with cardiac anaphylaxis. It is caused by inflammatory mediators released after exposure to drugs, food, environmental and other triggers. Oxidative stress occurring in various inflammatory disorders causes molecular damage with the production of advanced oxidation products (AOPPs) and advanced glycation end products (AGEs).

**Case presentation:**

Markers of oxidative stress were evaluated in a patient who had experienced KS after antibiotic administration in order to investigate the possible role of these molecules in KS. No data, up to now, are available on biomarkers of oxidative stress in patients with drug-induced KS.

**Conclusions:**

AOPPs, but not AGEs, were significantly increased in the KS affected patient compared to controls as already reported in mastocytosis affected patients.

## Background

Kounis syndrome (KS) is characterized by a concurrent occurrence of acute coronary syndromes with conditions associated with mast cell activation including allergic or hypersensitivity and anaphylactic or anaphylactoid insults [1].

The mechanism of KS consists in the release of inflammatory cytokines through mast cell activation, causing cardiac anaphylaxis with coronary artery vasospasms and/or atheromatous plaque erosion or rupture.

Three different variants of KS have been defined [2, 3]:

- Type I refers to patients with normal coronary arteries who develop coronary vasospasm after an allergic event. In these patients, electrocardiographic changes are secondary to ischemia. However, cardiac enzymes may or may not be normal.
  It is thought that this variant could be a manifestation of endothelial dysfunction or microvascular angina.
- Type II includes patients with a pre-existing coronary disease, in whom an allergic insult leads to plaque erosion or rupture, culminating in acute myocardial infarction or coronary vasospasm with normal cardiac enzyme and troponin levels.
- Type III includes patients with coronary artery stent thrombosis secondary to allergic reaction.

We report on a case of KS, occurred after antibiotic administration, that we investigated for serum levels of oxidative stress markers such as advanced oxidation protein products (AOPPs) and advanced glycation end products (AGEs) in order to evaluate the possible role of these molecules in KS.

Oxidative stress is involved in many inflammatory and immunologic diseases and it is the result of imbalance between endogenous production of free reactive oxygen species (ROS) and reduction of antioxidant defense mechanisms. As much as we know the formation of AGEs and AOPPs might be augmented in diseases linked to immune dysregulation. Among these, allergic pathologies are some of the most investigated [4]. Moreover, AOPP has been described to worsen heart functionality by influencing cardiac remodeling, determining cardiomyocyte apoptosis and cardiomyocyte death via Nox2/Rac1/superoxide-dependent TRAF3IP2/JNK signaling [5].

No data are available on biomarkers of oxidative stress in patients with drug-induced KS.

## Materials and methods

A 73-year-old woman, a smoker, with hypertension, dyslipidemia, post-surgical hypothyroidism, cancer of the bladder treated with excision, who had previously tolerated exposition to cephalosporines, presented with flushing, skin erythema, itching to the hands and lips, and chest pain after receiving ceftriaxone i.v. for a routine cystoscopy. Emergency treatment with Hydrocortisone 500 mg i.v. and chlorpheniramine 10 mg i.m. was administered. An electrocardiogram (ECG) was performed at the Emergency Unit which showed an acute myocardial infarction while cardiac enzymes and coronary angiography were normal revealing a case of type I drug-induced KS.

After experiencing antibiotic-induced KS, cardiovascular risk factors were evaluated; the patient was asked to stop smoking and statin anti-cholesterol treatment was prescribed.

Risk factors for anaphylaxis were evaluated such as serum basal blood levels of tryptase, total serum Immunoglobulin E levels and serum Immunoglobulin E measurements to beta-lactam antibiotics.

A serum sample was taken from the patient and also from 8 healthy volunteers as control.

Written informed consent, according to the declaration of Helsinki, was obtained from the patient and controls. Blood was withdrawn from the antecubital vein and allowed to clot at room temperature for 2 h. Serum was separated by centrifugation at 1200*g* for 15 min and stored at − 80 °C until used.

AOPPs and AGEs were measured by spectrophotometric and spectrofluorimetric methods, respectively.

For AOPP determination, 200 µl of blood serum was diluted 1:5 with phosphate-buffered saline (PBS) (pH 7.4- mM 0.01 M) and 200 µl of chloramines T (0–100 mol/l) for calibration, and 200 µl of PBS as blank were applied on a micro-titer plate. Ten microliters of 1.16 M Kl and 20 µl of acetic acid were added, and absorbance at 340 nm was measured immediately. The serum concentration of AOPPs was normalized to the total protein amount determined by the Bradford assay and expressed as chloramine nmoles/mg of protein.

For AGEs determination, serum was diluted 1:5 with PBS (pH 7.4- mM 0.01 M), and fluorescence intensity was recorded at maximum emission (∼440 nm) upon excitation at 350 nm and expressed in arbitrary units (AU). The serum concentration of AGEs was normalized to the total protein amount determined by the Bradford assay and expressed in AU for protein gram.

Each sample was analyzed in triplicate both for AOPPs and for AGEs determination.

## Statistical analysis

Data are expressed as medians. Differences between groups were analyzed by Mann–Whitney test. Correlation between two variables was evaluated with Spearman’s rho. The statistical analysis was performed with SPSS for Windows (version 17.0). The level of statistical significance was set at *P* < 0.05.

## Results

Serum basal blood levels of tryptase resulted within normal range (3.32 ng/ml), total Immunoglobulin E levels were 175 Ul/ml and serum Immunoglobulin E measurements to beta-lactam antibiotics, Penicilloyl G and V were 2.25 KU/l and 1.83 KU/l respectively.

As reported in Table 1, there was not a significant difference of the AGEs level between the patient, who presented a type 1 variant of Kounis syndrome with no underlying coronary artery disease, and the healthy controls. On the contrary, the serum level of AOPPs was significantly increased in the KS affected patient compared to controls.

**Table 1:** Concentration of AGEs (AU/g protein), AOPPs (nmol/mg protein) and control subjects in patients with KS

AGEs	109.58
AOPPs	3.643
Control for AGEs	135.775 ± 13.114
Control for AOPPs	0.804 ± 0.132

## Discussion

KS is quite rare, maybe underestimated, and can be fatal. Patients with an allergic reaction and concomitant symptoms which could suggest cardiac anaphylaxis, should be monitored from both an allergic and cardiologic point of view, taking into account a possible case of KS [6].

KS was first described by Kounis and Zavras in 1991 as an allergic angina syndrome [7].

It was then reported that KS is not limited to angina symptoms, but could progress to acute myocardial infarction and stent thrombosis with occluding thrombus, caused by inflammatory mediators such as histamine, platelet activating factor, cytokines, chemokines, tryptase, chymase, prostaglandins and leukotrienes [8].

Mast cells identified in human heart, between myocardial fibers, in the perivascular tissue, in the adventitia and arteria intima, express high affinity immunoglobulin E (IgE) receptors (FcεRI). The involvement of these mediators might explain the role of cardiac mast cells in systemic and cardiac anaphylaxis. Thus, the human heart can be considered a site and target of anaphylaxis [9].

It has been shown both in vitro and in vivo that oxidative stress activates mast-cells’ degranulation. Experimental data on the oxidative status of mice heart after cardiac anaphylaxis, induced by the injection of ovalbumin in the aortic cannula, showed that nitric oxide (NO) may not be the only influential mediator of redox changes in cardiac anaphylaxis [10]. Mastocytosis, a clinically heterogeneous disease characterized by clonal proliferation of mast cells, which can cause systemic anaphylaxis with possible cardiac involvement [11], is associated with a state of increased oxidative stress with high serum levels of AOPPs likely consequent to an increased ROS production from mast cells, without significant changes in circulating concentrations of AGEs [12]. The latter are a biomarker known to be intimately involved in the pathophysiology of cardiovascular diseases [13] and associated with a higher thrombotic risk [14]. Since ROS are almost impossible to measure directly, oxidative stress values could be indirectly measured by dosing advanced oxidation protein products (AOPPs) and advanced glycation end products (AGEs). These markers of oxidative stress were evaluated in subjects affected by mastocytosis with a positive correlation to mast cell activation [12].

**Fig. 1:**
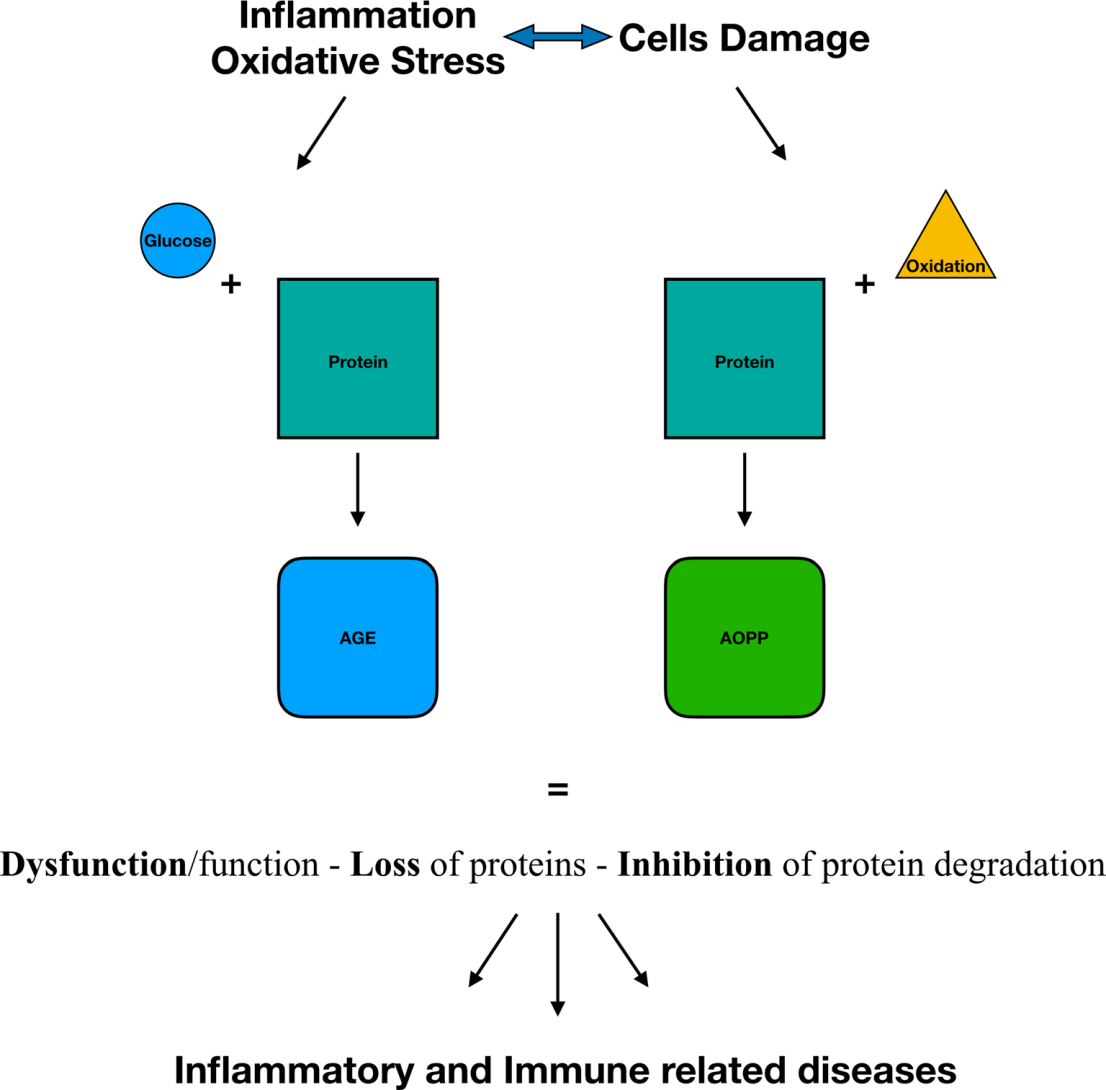
Physiopathology mechanism of oxidative stress bringing to the production of AOPPs (advanced oxidation products) and AGEs (advanced glycation end products)

The results obtained by the serum analysis of the KS affected patient seem to indicate a sort of analogy between Mastocytosis and KS; in fact, the higher levels of AOPPs compared to controls confirm once more the fundamental role acted by mast cells in KS. In what measure and how frequently oxidative stress is associated to this syndrome it has to be clarified. Basing our future researches on these results, it could be a reliable objective to investigate a wider number of samples obtained by KS patients. Both AGE and AOPP (Fig. 1), augmented in diseases linked to immune dysregulation, could be in a near future standardized as a routine exam such as CRP and ESR; in fact these techniques are cheap, fast, and easy to execute. Therefore, further studies could also lead us to screening patients for a higher risk of disease by a simple and low cost biomarker dosage.
